# Unravelling the Pathogenetic Mechanisms in Congenital Aortopathies: Need for an Integrative Translational Approach

**DOI:** 10.3390/jcm9010204

**Published:** 2020-01-11

**Authors:** Evaldas Girdauskas, Harald Kaemmerer, Yskert von Kodolitsch

**Affiliations:** 1 Department of Cardiovascular Surgery, University Heart and Vascular Center Hamburg, Martinistraße 52, 20246 Hamburg, Germany; 2 Partner site Hamburg/Lübeck/Kiel, German Center of Cardiovascular Research (DZHK), 20246 Hamburg, Germany; kodolitsch@uke.de; 3 Department of Congenital Heart Disease and Pediatric Cardiology German Heart Center Munich, Technical University Munich, 80333 Munich, Germany; Kaemmerer@dhm.mhn.de; 4 Department of Cardiology, University Heart and Vascular Center Hamburg, 20246 Hamburg, Germany

**Keywords:** aortopathy, bicuspid aortic valve, congenital heart disease

## Abstract

Congenital heart disease (CHD)-associated aortopathy is a very heterogeneous entity with a wide spectrum of clinical presentations. The pathogenesis of aortopathy is still incompletely understood, and, therefore, the best prevention and management strategy is currently unknown. The most common entity of CHD-associated aortopathies is bicuspid aortic valve (BAV)-associated aortic disease (so called bicuspid aortopathy) that is found in 50%–60% of BAV individuals. BAV aortopathy has been reported in association with an increased risk of aortic events, especially aortic dissection and sudden cardiac death. Risk stratification of adverse aortic events is still very rudimentary and considers only the maximal aortic diameter, which makes it unsuitable for an individual risk prediction. This introductory Editorial highlights the unmet clinical need for more integrative and translational research to unravel pathogenetic pathways in the development of CHD-associated aortopathies, integrating recently identified genetic lesions and knowledge on circulating biomarkers and microstructural changes in the diseased aorta.

## 1. Introduction

Congenital and hereditary heart disease is a very heterogeneous entity with a wide spectrum of clinical presentations. One of the most important complications in the natural as well as in the postinterventional or postoperative course is congenital heart disease (CHD)-associated aortic disease (so called aortopathy).

This editorial describes an integrative multidisciplinary approach to systematically addressing the pathogenetic pathways in the development of CHD-associated aortopathies, with a special focus on bicuspid aortopathy. An integrative translational approach that covers the whole range of pathogenetic pathways from the target genetic signaling, circulating microRNAs to the effector microstructural lesions in the diseased aortic wall and aortopathy progression is needed to stimulate multidisciplinary research in CHD-associated aortopathies.

The pathogenesis of CHD-associated aortopathies is still incompletely understood, and thus, the best diagnostic and treatment strategies are currently unknown. Given the fact that bicuspid aortic valve (BAV) is the most common congenital anomaly of the human heart (i.e., 0.5%–1.35% prevalence in the general population [1]), BAV-aortopathy represents the most frequent form of CHD-associated aortic disease [2].

BAV development is characterized by the lack of splitting of two adjacent cusps during embryogenesis, with a variable degree of the persisting congenital fusion (raphe) [3]. BAVs are organized according to the Sievers classification based on the type of fusion, raphe, and aortic valve lesion [4]. More than 50% of BAV patients have a dilated proximal aorta (i.e., bicuspid aortopathy) which has been linked to an increased risk of aortic dissection or rupture [5]. The risk of aortic complications is extrapolated from the maximal aortic diameter. Usually, a larger aorta is associated with a higher risk of an aortic event, while the risk increases considerably with an ascending aorta diameter ≥60 mm or ≥4.25 cm/m^2^ [6]. Therefore, the current guidelines for prophylactic aortic surgery are based on an aortic diameter of 50–55 mm. However, more than 90% of aortic dissections occur with an aortic diameter smaller than 50–55 mm and therefore cannot be prevented by following those guidelines [7]. Therefore, the maximum aortic diameter alone is not sufficient for risk stratification and prophylactic surgery in bicuspid aortopathy. The identification of novel biomarkers (i.e., specific genetic variants and/or serologic biomarkers) involved in the pathogenetic pathways underlying bicuspid aortopathy is needed.

We aim to discuss recently published data on bicuspid aortopathy to unravel pathogenetic pathways in the development of CHD-associated aortopathies, integrating recently identified genetic lesions and knowledge on circulating biomarkers and microstructural changes in the diseased aorta.

## 2. Genetic Signaling Pathways and Bicuspid Aortopathy

The *NOTCH1* signaling pathway plays a crucial role in a variety of intercellular processes, including angiogenesis and cardiac valve development and differentiation. Specifically, *NOTCH1* has a role in the migration of cells from the cardiac cushions and cardiac jelly into the conotruncal cushions, from which the aortic and pulmonary valves are formed [8]. Alterations in the *NOTCH1* pathway have been previously reported in the pathogenesis of CHD-associated aortopathies and, specifically, in BAV development [9,10,11]. McKellar and coauthors were the first to report the association between rare non-synonymous *NOTCH1* missense variants and BAV with ascending aortic aneurysms [9]. In a cohort of 48 patients with concomitant BAV and ascending aortic aneurysms, they identified four *NOTCH1* missense variants in five (10.5%) patients, which were significantly more frequent as compared to the control subjects (i.e., 3/144, 2.1%). The authors hypothesized that the interaction between the *NOTCH1* signaling pathway (valvulogenesis) and *TGFß* (aortic extracellular matrix regulation) makes BAV patients with *NOTCH1* mutations more susceptible to aortic aneurysm formation.

In accordance to these findings, subsequent studies showed that the prevalence of *NOTCH1* missense variants was in the range of 10%–18% in BAV patients with concomitant ascending aortic aneurysms (see Table 1) [10,11,12]. These potentially deleterious *NOTCH1* variants were mostly absent in the tricuspid aortic valve (TAV) control groups without aortopathy.

These potentially pathogenic *NOTCH1* mutations are situated within strategic domains responsible for ligand binding. These variants are likely to alter *NOTCH1* function by interfering with its capability to build disulfide bonds or by disrupting post-translational processing. In line with these findings, in silico prediction tools also indicated deleterious effects of these *NOTCH1* variants. In our previous study, we identified five rare *NOTCH1* variants that significantly changed *NOTCH1* protein structure and affected predominantly the epidermal growth factor (EGF)-like domains. Changes in *NOTCH1* protein structure can impair its ligand binding ability [13].

Another recent study revealed significantly lower levels of circulating NOTCH1 as well as lower expression of genes encoding components and ligands of the *NOTCH1* pathway in the aortic tissue in BAV subjects as compared to TAV subjects, irrespective of the presence of ascending aortic aneurysm [14].

In summary, there is an emerging evidence from the recent literature of the pathogenetic impact of *NOTCH1* mutations in the development of bicuspid aortopathy. Such genetic alterations could be found in every 10th patient presenting with BAV and simultaneous ascending aortic aneurysm.

## 3. Circulating microRNAs and Bicuspid Aortopathy

Several previous studies demonstrated the downregulation of circulating microRNAs in progressive vasculopathies [15,16]. Wu et al. revealed an impaired expression of miR-17-related miRNAs (miR-17, miR-18a, miR-19a/b, miR-20a/b, miR-106a/b, and miR-93) in the aortic tissue, which was associated with reduced tissue inhibitor of matrix metalloproteinases (TIMP) activity and concomitant overexpression of matrix metalloproteinase-2 (MMP2) [15]. The authors hypothesized that an altered miR-17 expression impacts TIMP/MMP homeostasis and thereby induces aortopathy progression.

Recent studies revealed a significant association between downregulation of specific microRNAs and bicuspid aortopathy [17,18,19]. Martinez-Micaelo and coauthors applied a microarray approach to examining plasma microRNAs which could be specific for BAV and aortopathy [19]. They found that the expression levels of circulating miR-122, miR-130a, and miR-486 were significantly associated with aortic valve morphology (i.e., bicuspid vs. tricuspid), whereas the downregulation of circulating miR-718 strongly correlated with the proximal aortic diameter and ascending aortic dilation. Our group found a significant downregulation of blood miR-17 and miR-106a in a BAV aortopathy cohort, as well as a strong correlation between aortic root size and blood levels of miR-17 and miR-106a in a pooled cohort of 96 BAV patients [20].

Furthermore, we focused specifically on those patients who underwent an isolated aortic valve surgery due to BAV and had a significant aortopathy progression during the postoperative follow-up (i.e., increase of ascending aortic diameter ≥ 3.0 mm). We found significantly lower values of circulating miR-17, miR-106a, and miR-145 in a BAV cohort with aortopathy progression vs. a cohort with unchanged aortic diameters (i.e., aortic diameter increase <3 mm) [21]. A significant inverse linear correlation (r = −0.48, *p* = 0.03) was revealed between blood miR-145 levels and proximal aortic diameter in BAV patients who underwent an isolated aortic valve surgery [21].

MiR-145 has the capability to modify vascular smooth muscle cell (VSMC) phenotype and thereby impact the progression of vasculopathies [22,23]. Circulating miR-145 has been also shown to alter VSMCs’ phenotype from a proliferative to a contractile state [23]. MiR-145-mediated VSMCs’ phenotypic switch and its mediated impact on neointimal formation have been recently demonstrated in several experimental designs [24,25].

## 4. Genetic Signaling Pathways and Circulating microRNAs

Boucher et al. were the first to describe the interaction between miR-145 levels and *NOTCH* signaling in VSMCs [26]. The authors hypothesized that an upregulation of the Jag-1/NOTCH signaling pathway leads to increased miR-145 levels, thereby inducing the VSMC contractile phenotype [26]. Opposite to that, a decrease in *NOTCH* signaling would lower the levels of circulating miR-143 and miR-145. By implementing multiple experimental steps, the authors convincingly demonstrated that *NOTCH* signaling requires the induction of miR-143/145 to promote the VSCM contractile phenotype. A close interaction between microRNA-145 expression and *NOTCH* signaling has been recently demonstrated in the mediation of glioma cells apoptosis [27].

We could demonstrate a strong correlation between previously reported *NOTCH1* gene missense mutations and circulating miR-145 levels, in that miR-145 expression was significantly decreased in a cohort with *NOTCH1 (+)* variants [21]. Rare *NOTCH1* variants may deleteriously change the protein structure and thereby reduce basal *NOTCH* signaling, which in turn will alter the expression of circulating miR-145 and cause aortopathy progression [21].

## 5. Microstructural Changes and BAV Aortopathy

The most remarkable histological features in the ascending aorta of BAV patients are the immaturity of VSMCs [28] and the limited presence of ageing histopathology features, such as inflammation and cystic medial degeneration (CMD) [29], as compared to the ascending aortas of TAV patients. Grewal and coauthors found a significantly lower expression of α smooth muscle actin (αSMA), smooth muscle 22α, and calponin and an almost absent expression of smoothelin in patients with BAV [28]. The authors hypothesized that there might be a defective VSMC differentiation in BAV patients, which is possibly linked to decreased lamin A/C expression. In accordance to these findings, another research group demonstrated accentuated VSMC apoptosis and MMP-9 overexpression in the proximal aortas of BAV patients [30]. Quite different, the proximal aorta of TAV patients was characterized by a more advanced elastic fragmentation, cystic medial necrosis, medial fibrosis, and inflammation, indicating specific pathogenetic mechanisms in aortopathy genesis in BAV vs. TAV patients [31].

## 6. Integration of Genetic Signaling Pathways, Circulating microRNAs, and Histological Changes in BAV Aortopathy

Considering the previously described modulation of the contractile VSMC phenotype by *NOTCH* signaling and the associated miR-143/145 expression [26,27], we hypothesized that the observed effector “immaturity” or dedifferentiation of the proximal aortic wall in BAV aortopathy might be transmitted by a defective *NOTCH1* signaling and the subsequent down-regulation of miR-145 expression. Several experimental studies seem to support this hypothesis [23,31]. The expression of VSMC differentiation marker genes (αSMA, calponin, and smooth muscle myosin heavy chain (SM-MHC)) was upregulated using premiR-145 or adenovirus-expressing miR-145 (Ad-miR-145) but were downregulated by adding the miR-145 inhibitor 2’OMe-miR-145 [23]. Another experimental study by Tang et al. showed that NOTCH intracellular domain (Notch ICD) may form a complex with C-promoter-binding factor-1 (CBF-1), which directly induces αSMA expression [31]. Using primary human VSMCs, these authors demonstrated that the expression of the constitutive active intracellular domain of human NOTCH1, NOTCH2, or NOTCH4 receptors significantly increased αSMA levels. This study confirmed that *NOTCH* signaling determined the expression of VSMC differentiation markers including αSMA [31].

## Figures and Tables

**Table 1:** *NOTCH1* mutations published in association with bicuspid aortic valve (BAV) and aortopathy.

Authors Group	Publication Year	Patients (*n*)	Overall Prevalence *NOTCH1* Variants	Specific *NOTCH1* Variants
Mohamed et al. [11]	2006	48	8.3%	p. T596M p. P1797H p. P1377S p. V2285I
McKellar et al. [9]	2007	48	10.5%	p. A1343V p. P1390T p. R1350L p. P1377S
Foffa et al. [10]	2013	11	18.2%	p. P284L p. Y1619X
Girdauskas et al. [12]	2017	63	9.5%	p. T445M p. R621H p. K1498E p. P1390T p. A1343V
